# Are We Drifting in Our Ethics?

**Published:** 1895-08

**Authors:** 


### *EDITORIAL.*

---

The office of THE JOURNAL is in room 330 Equitable Building.

The editors are not responsible for views expressed by contributors.

Articles for publication should reach this office not later than the 15th instant.

Address all communications, and make all remittances payable to THE ATLANTA MEDICAL AND SURGICAL JOURNAL, Atlanta, Ga.

Reprints of articles will be furnished, when desired, at a nominal cost. Requests for the same should always be made *on the manuscript.*

---

### ARE WE DRIFTING IN OUR ETHICS?

The inquiry, "Where are we at?" is said to be not so important as "Whither are we going?" This seems to be the idea of Dr. Leartus Connor, of Detroit, who has lately written an excellent paper, entitled "Drifting: Who, How, and Whither." (Bulletin of the American Academy of Medicine.)

Dr. Connor traces the history of certain gradual changes in sentiment in relation to our medical ethics since the adoption of the written code, nearly fifty years ago. The growing desire on the part of some not to be hampered by the restrictions embodied in the law of 1847 first culminated in official action in the New York State Medical Society, several years ago. This society "abrogated the written law, and returned to the unwritten law," which, we are told, represented the "first principles of the medical profession—principles which satisfied the profession during thousands of years," the reformers preferring the larger individual liberty accorded them under the old régime. The Mississippi Valley Medical Association imposes no restrictions upon members in the matter of consultations; neither do some of the medical societies of St. Louis Chicago, and other cities.

"The drifting is further seen in that regulars and sectarians work side by side on boards of health, State and local, and on boards of other State institutions. This very imperfect sketch shows that, whether we like it or not, the fact cannot be disguised that the professional relationships of regulars and irregulars are, at many points, multiplying and extending. If, finally, through these relationships the sectarians shall be swallowed up by the medical profession, history will simply repeat itself.

"Whither does this drifting tend? The history of the past and the logic of existing social forces point to but one result—viz., the absorption of all ideas that are true and helpful, and all persons who are honest and of good report, into the medical profession. To this end larger liberty will be accorded the individual physician. He will be permitted to choose his professional associates from among those whom he knows to be properly educated, legally qualified to practice medicine where they reside, and of good report. Qualifications other than these will be regarded as purely local in their necessity and temporary in their existence, and to be controlled entirely by the individual physician. It were wiser to object to a consultation with a physician because of known ignorance, lack of skill, dishonest methods, disreputable character, than because of his sectarian name. Objection on the first ground is readily understood by any layman, but objection on the second fails to commend itself to most persons, and not infrequently brings the objecting physician into discredit."

